# Dining in Tuva: Social correlates of diet and mobility in Southern Siberia during the 2nd–4th centuries CE

**DOI:** 10.1002/ajpa.24506

**Published:** 2022-03-07

**Authors:** Marco Milella, Gino Caspari, Zita Laffranchi, Gabriele Arenz, Timur Sadykov, Jegor Blochin, Marcel Keller, Yulija Kapinus, Sandra Lösch

**Affiliations:** ^1^ Department of Physical Anthropology, Institute of Forensic Medicine University of Bern Bern; ^2^ Department of Archaeology University of Sydney Sydney Australia; ^3^ Institute of Archaeological Sciences University of Bern Bern Switzerland; ^4^ Institute for the History of Material Culture Russian Academy of Sciences St. Petersburg Russia; ^5^ Estonian Biocentre, Institute of Genomics University of Tartu Tartu Estonia; ^6^ Volga‐Ural Center for Paleoanthropological Research SSSPU Samara Russia

**Keywords:** carbon, millet, nitrogen, sulfur, Tunnug

## Abstract

**Objectives:**

Contemporary archeological theory emphasizes the economic and social complexity of Eurasian steppe populations. As a result, old notions of “nomadic” cultures as homogenously mobile and economically simple are now displaced by more nuanced interpretations. Large part of the literature on diet and mobility among Eurasian pastoralists is focused on the Bronze and Iron Ages. The underrepresentation of more recent contexts hampers a full discussion of possible chronological trajectories. In this study we explore diet and mobility at Tunnug1 (Republic of Tuva, 2nd–4th century CE), and test their correlation with social differentiation.

**Materials and Methods:**

We compare demographic patterns (by age‐at‐death and sex) of carbon, nitrogen, and sulfur stable isotope ratios (δ^13^C, δ^15^N, and δ^34^S) among 65 humans and 12 animals from Tunnug1 using nonparametric tests and Bayesian modeling. We then compare isotopic data with data on perimortal skeletal lesions of anthropic origin and funerary variables.

**Results:**

Our analyses show that: (1) diet at Tunnug1 was largely based on C_4_ plants (likely millet) and animal proteins; (2) few individuals were nonlocals, although their geographic origin remains unclarified; (3) no differences in diet separates individuals based on sex and funerary treatment. In contrast, individuals with perimortal lesions show carbon and nitrogen stable isotope ratios consistent with a diet incorporating a lower consumption of millet and animal proteins.

**Discussion:**

Our results confirm the previously described socioeconomic variability of steppe populations, providing at the same time new data about the economic importance of millet in Southern Siberia during the early centuries CE.

## 1 INTRODUCTION

### 1.1 Historical and archeological background

The economic complexity of Eurasian steppe populations is increasingly becoming a topic of interest in archaeology and anthropology. The dismissal of simplistic analytical models (“nomadic bias”—Spengler et al., 2021) have stimulated, especially in the last decade, a rich research agenda. Aspects traditionally assumed as defining for these cultures (dependency on domesticated mammals, low social complexity, and high mobility) are nowadays substituted by a perspective which stresses their heterogeneity, economic flexibility and social complexity (Frachetti, 2009; Frachetti et al., 2010; Honeychurch, 2014; Honeychurch & Makarewicz, 2016; Spengler, Cerasetti, et al., 2014; Spengler, Frachetti, et al., 2014; Spengler et al., 2021; Ventresca Miller & Makarewicz, 2019). Current archeological research on Eurasian societies has repeatedly highlighted such ecological flexibility during prehistory (e.g., Frachetti, 2009; Frachetti et al., 2010; Spengler, 2015; Spengler, Cerasetti, et al., 2014; Spengler, Frachetti, et al., 2014; Wright et al., 2009). Besides archeological, archaeozoological and palaeobotanical investigations, isotopic analyses of ancient human biological tissues (bones, teeth, and hairs) are continuously providing new data about past dietary and mobility patterns in these populations (Hanks et al., 2018; Lightfoot et al., 2015; Murphy et al., 2013; Svyatko et al., 2007, 2013; Svyatko, Polyakov, et al., 2017; Svyatko, Reimer, & Schulting, 2017; Ventresca Miller et al., 2017, 2018, 2019, 2021; Ventresca Miller & Makarewicz, 2019). Isotopic studies of Eurasian steppe populations have been focused, to a large extent, on the reconstruction of diet and, to a lesser extent, on mobility of Bronze and Iron Age populations from the Pontic region, through Kazakhstan to Southern Siberia and Mongolia. Despite their geographical heterogeneity, these studies have repeatedly reached similar conclusions: (1) diets of these communities often incorporated resources like freshwater foodstuffs (e.g., freshwater fish) and domesticated grains (e.g., millet) (Hollund et al., 2010; Lightfoot et al., 2015; Motuzaite Matuzeviciute et al., 2015; Murphy et al., 2013; Svyatko et al., 2013; Ventresca Miller & Makarewicz, 2019; Zhang et al., 2015); (2) mobility was mostly related to seasonal transhumance, while long‐distance movements were much rarer (Machicek et al., 2019; Ventresca Miller et al., 2021).

The need for a conceptual and terminological re‐evaluation of traditional categories has been recently stressed by Spengler et al. (2021) in their synthesis of the available archaeobotanical, archeological, biogeochemical, and zooarchaeological evidence for Eastern Central Asia between the second and first millennia BCE. This work emphasizes how large parts of the population living in the analyzed contexts carried a mixed economy of farming and herding (agro‐pastoralism), rather than specialized mobile pastoralism (nomadism).

The above considerations bear on our understanding of the emergence, degree, and expression of social complexity and social differentiation among agro‐pastoralist societies. A traditional perspective envisages pastoral groups as economically constrained and egalitarian (Salzman, 2004), and social complexity as evolving in parallel with reliance on farming (Khazanov, 1978). Based on the multifaceted economy of steppe populations, however, such dichotomist framework appears increasingly incapable to capture the nuances of social differentiation among these groups.

Only few studies have explicitly tested the possible intersection between social complexity, diet, and mobility among Eurasian societies by means of a combined analysis of anthropological, isotopic, and archeological data. Moreover, whereas isotopic studies have especially focused on Bronze and Early Iron Age contexts, relatively less data (i.e., Kradin et al., 2021; Ventresca Miller & Makarewicz, 2019; Wilkin et al., 2020) are available for the first centuries CE.

In this work, we contribute to biocultural reconstructions of diet, mobility, and social complexity among Eurasian steppe populations by analyzing the relationship between isotope, demographic, and archeological data in a funerary context from Southern Siberia dating to the early centuries CE (Tunnug1, Republic of Tuva, 2nd–4th c CE).

### 1.2 Geographic and archeological context

**FIGURE 1:**
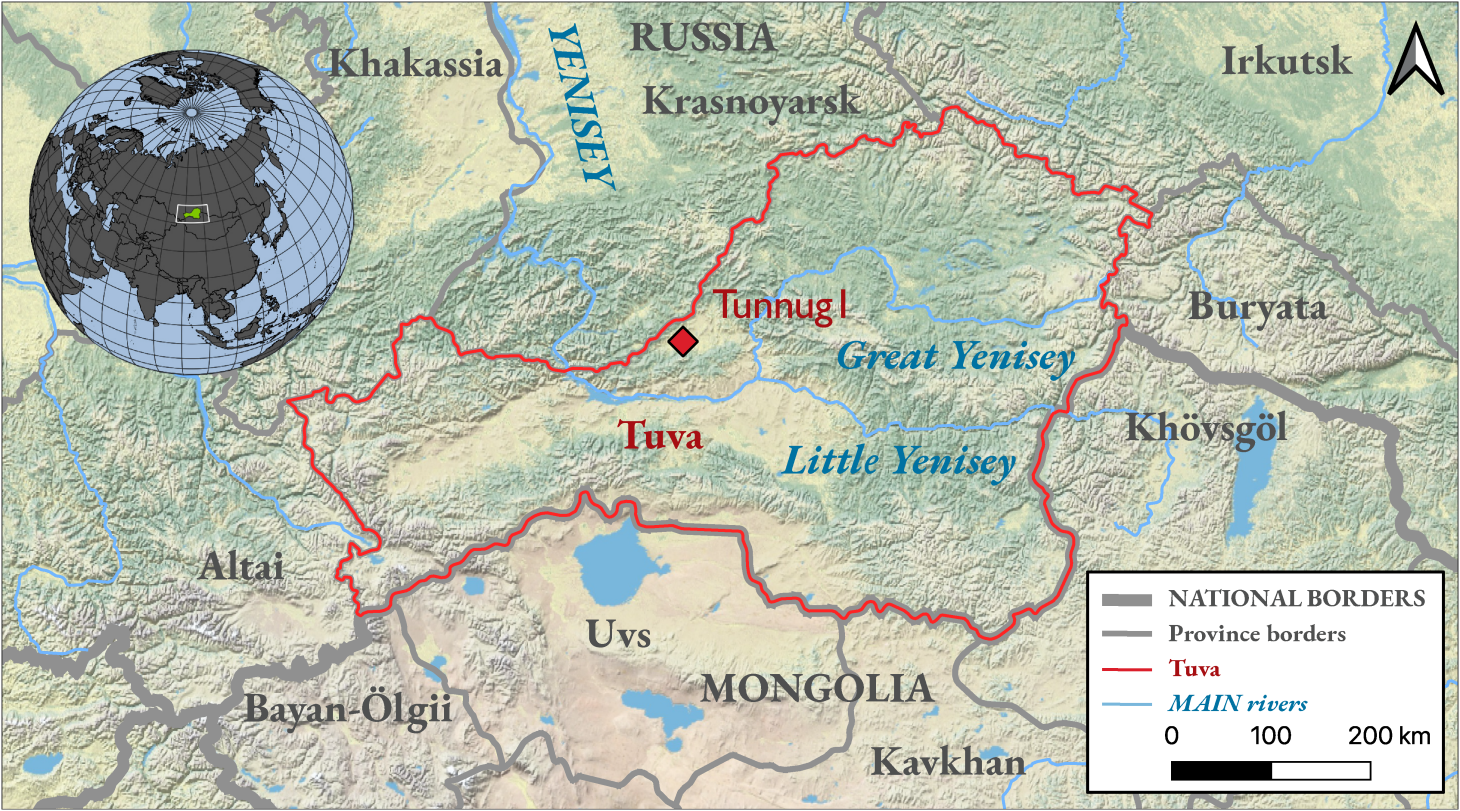
Geographic position of Tunnug1 (red diamond). Projection: WGS84/pseudo‐Mercator. Made in QGIS3.12 using ESRI physical as basemap and natural earth (1:10) for rivers and borders

The territory of Tuva Republic is located in Southern Siberia, Russia. Situated between the Sayan mountains in the North, the Altai in the West and separated from the Eastern steppe by the Tannu‐Ola mountain range, Tuva features a continental climate (−50°C in winter and up to 40°C in summer) and a semi‐arid environment characterized by a mixture of east Siberian Taiga, Mongolian steppe, and semi‐desert elements (Chugunov et al., 2010). Through the Yenisei, whose tributaries Little and Great Yenisei confluence at Tuva's modern capital Kyzyl, Tuva is connected to the Minusinsk Hollow in the North (Figure 1). The region plays an important role in the social development of late prehistoric steppe societies, as it features some of the earliest and largest burial mounds dating to the Early Iron Age (9th c. BCE) (Caspari, 2020). The construction of these monuments coincides with and is evidence for the development of steeply hierarchical societies and elites in a marginal environment. Investigations of monumental burial mounds have yielded insights into the dynamics associated with the so‐called Scythian material culture and the transfer of new ideas across large stretches of Inner Asia (Caspari et al., 2018, 2019; Gryaznov, 1980; Parzinger, 2006: 606ff; Sadykov et al., 2020). With the expansion of the first steppe Empire in the 2nd c. BCE, Tuva sees an amalgamation of cultural characteristics and stylistic elements of Xiongnu material culture with indigenous elements, showing long‐distance connectivity of networks and a degree of integration into the social system of the empire including specific burial traditions and prestige goods (Kilunovskaya & Leus, 2018, 2020a, 2020b; Leus, 2011; Leus & Bel'skiy, 2016; Miller, 2011). The centuries after the decline of the Xiongnu Empire see the emergence of the distinct local Kokel culture (Sadykov et al., 2021) lasting from the 2nd c. to the 5th c. CE.

The excavation of Tunnug1 is a joint Russian‐Swiss project focusing on the investigation of a monumental Early Iron Age burial mound dating to the 9th c. BCE (Caspari et al., 2020). Tunnug1 is located in the Uyuk Valley, which features a semi‐arid landscape sheltered by mountains and ideal for herding of sheep, goat, and horses. The Uyuk Valley, draining into the Greater Yenisei, in its northwest side, is closed off by the western Sayan Mountains, while in the south it is separated from southern Tuva by the Uyukskij Mountains (Figure 1).

Geophysical and remote sensing surveys revealed an extensive periphery of the main burial mound with stone structures indicating a long‐term funerary use of the site (Caspari et al., 2018). An amorphous stone structure and several individual burial and ritual features of the Kokel culture affiliation are located in the southern periphery. These structures yielded well‐preserved human remains (see Milella et al., 2021) which are the basis of this study. A large number of these individuals (22/87: 25.3%) present perimortem skeletal lesions, mostly at the level of the neck and head. The position and typology of these traumas suggest their occurrence in the contexts of face‐to‐face combat and, possibly executions (Milella et al., 2021). The dynamics of these violent events and the identity of these individuals (population affiliation, social status, and geographic origin) remain, however, unclear.

### 1.3 Stable isotopes of carbon, nitrogen, and sulfur (δ^13^C, δ^15^N, and δ^34^S): Diet and mobility

Stable isotope ratios of carbon (δ^13^C) and nitrogen (δ^15^N) from bone collagen are routinely used for the reconstructions of ancient human diet (e.g., Ambrose & Deniro, 1986; Laffranchi et al., 2016; Lösch et al., 2006; Milella et al., 2019; Schoeninger & Moore, 1992).

Carbon isotope ratios (δ^13^C) in bone collagen largely reflect the protein component of the diet, although being also influenced by carbohydrates and lipids (Fernandes et al., 2012; Froehle et al., 2010; Howland et al., 2003). The δ^13^C values reflect the relative proportion in the diet of plants featuring different photosynthetic pathways in the diet (C_3_ vs. C_4_ plants) (Deniro & Epstein, 1981; Van der Merwe, 1982), and may also provide information about past environmental and climatic conditions (Laffranchi et al., 2016; Siebke et al., 2020; Van Klinken et al., 2002).

δ^15^N undergoes a significant enrichment for each trophic level in a food chain (3‰–6‰) (Hedges & Reynard, 2007; O'Connell et al., 2012). Accordingly, δ^15^N values in bone collagen reflect the trophic level of an organism, and allow the estimation of the relative amount of animal and vegetal proteins in their diet (Ambrose, 1991; Deniro & Epstein, 1981; Hedges & Reynard, 2007).

Sulfur isotope ratios (δ^34^S) in bone collagen are influenced by the local bedrock and atmospheric composition, and show a small isotopic offset (on average +0.5 ± 2.4‰) by trophic level (Nehlich, 2015). Average δ^34^S values tend to differ between terrestrial and marine environments, with the former presenting a range between −10‰ and +20‰ and marine water being closer to +20‰ (Nehlich, 2015). This feature explains the use of δ^34^S values for the test of marine resource contribution in the diet. δ^34^S values in freshwater ecosystems are quite variable (Nehlich, 2015) but, in those cases where they differ from terrestrial ecosystems, they provide useful information about the dietary exploitation of these biomes (Nehlich et al., 2011; Privat et al., 2007). The close correlation between δ^34^S in bone collagen and local geology, and the small fractionation along the food webs suggest the suitability of sulfur isotope ratios when estimating regional mobility among past populations. The use of δ^34^S in mobility research is relatively unexplored (compared with the use of oxygen and strontium), but promising, as suggested by the few studies which applied this approach on archeological materials (Cheung et al., 2017; Moghaddam et al., 2014; Paladin et al., 2020; Vika, 2009).

### 1.4 Aim of the study and hypotheses

In this study, we combine demographic (sex and age‐at‐death), isotopic (stable isotope ratios of carbon, nitrogen, and sulfur), paleopathological (patterns of trauma) and archeological (presence–absence of grave goods items) data from the Kokel burials of Tunnug1 and explore the possible association between diet, mobility, violence and funerary treatment.

Specifically, we test the following hypotheses:

Assuming a mixed subsistence economy for the population under study (see Murphy et al., 2013; Spengler et al., 2021; Ventresca Miller et al., 2021; Ventresca Miller & Makarewicz, 2019), we expect the diet of this community to be represented by a heavy reliance on animal proteins coupled with other resources (C_4_ plant products and/or freshwater fish).

According to previous archeological and ethnographic studies (Ventresca Miller et al., 2021) we expect mobility at Tunnug1 to be relatively low and represented by few nonlocal individuals.

Based on the low variability in funerary customs at Tunnug1 (Milella et al., 2021; Sadykov et al., 2021), we expect a relatively low degree of social differentiation, and minor to no differences in diet based on sex or social status in this community.

## 2 MATERIAL AND METHODS

### 2.1 Biological profile

The human sample includes 65 individuals representing both sexes and different age classes (Table 1). A detailed demographic analysis of Tunnug1 has been previously published by Milella et al. (2021). In brief, we estimated subadult age‐at‐death based on the development and eruption of deciduous and permanent dentition, diaphyseal measurements, and degrees of epiphyseal fusion (AlQahtani et al., 2010; Maresh, 1970; Moorrees et al., 1963; Ubelaker, 1989). For the estimation of adult age‐at‐death, we used the morphological changes of the symphysis of the pubis, the auricular surface of the ilium, and sternal ends of the ribs (Brooks & Suchey, 1990; Buckberry & Chamberlain, 2002; Iscan et al., 1984). We then grouped all individuals in seven age classes: neonates (up to 3 months of age), infants (4 months–3 years), children (3–12 years old), adolescents (13–18 years old), young adults (19–34 years old), middle adults (35–49 years old), and old adults (≥50 years old). In cases where a more accurate estimation of age was not possible (e.g., due to poor preservation of the remains) we used two broad classes: subadults (ca. <19 years old) and adults (ca. ≥19 years old).

**TABLE 1:** Distribution of the sample by age‐at‐death and sex

Age class	Age range	Tot	%total	F	%age class	M	%age class	NA	%age class
Neonate	0–3 months	3	4.6	0	0.0	0	0.0	0	0.0
Infant	4 months–3 years	9	13.8	0	0.0	0	0.0	0	0.0
Child	3–12 years	11	16.9	0	0.0	0	0.0	0	0.0
Adolescent	13–18 years	3	4.6	0	0.0	0	0.0	0	0.0
Young adult	19–34 years	13	20.0	3	23.1	8	61.5	2	15.4
Middle adult	35–49 years	8	12.3	1	12.5	7	87.5	0	0.0
Old adult	≥50 years	10	15.4	3	30.0	6	60.0	1	10.0
Adult	≥19 years	8	12.3	0	0.0	0	0.0	8	100.0

We estimated sex only for adults, based on the dimorphic features of the innominate bone, the cranium, and mandible (Ascadi & Nemeskeri, 1970; Ferembach et al., 1980; Phenice, 1969).

### 2.2 Carbon, nitrogen, and sulfur stable isotopes

#### 2.2.1 Sample preparation

For the analyses of stable isotope ratios from bone collagen of humans, we sampled portions of femoral diaphysis or—if missing—other long bones, ribs or cranial elements (Table S1). We collected 12 animal bone samples to be used for reconstructing the local trophic baseline. These include two bovids, two canids, one equid, and seven caprinae (goat/sheep). Most of the animal samples come from the Kokel phase of Tunnug1. One sheep/goat pertains to a later (Medieval) burial (Object 37).

For the analyses, caprinae and bovids were grouped in the “herbivore” group. We decided to exclude the horse from the latter group since horses usually show isotopic values deviating from those of ruminants. Differences in digestive processes between foregut and hindgut fermenters may indeed result in different isotopic values (Hanks et al., 2018; Ventresca Miller et al., 2018). The extraction was performed following an acid–base–acid extraction method modified after Ambrose (1990, 1993), Deniro (1985), and Longin (1971). After cleaning with distilled water, all samples were pulverized in a mix miller at 20 bps for 60 s. Then, 500 mg ± 3 mg of bone powder was demineralized with 10 ml of 1 M hydrochloric acid (HCl) for 20 min at room temperature. The solution was washed until neutral (pH ~ 6–7). About 10 ml of 0.125 M of sodium hydroxide (NaOH) was added and left for incubation at room temperature for 20 h. The solution was then washed until neutral, and 10 ml of 0.001 M HCl was added. The samples were placed in a water bath for incubation at 90°C (10–17 h). The solubilized collagen was filtered (VitraPOR filter‐funnel, porosity 16–40 μm) and lyophilized at 0.42 mbar for a minimum of 48 h. Of each sample, three times 3.0 mg ± 0.3 mg collagen was weighed into tin capsules.

#### 2.2.2 Mass spectrometric analysis

The isotope ratios of carbon (^13^C/^12^C), nitrogen (^15^N/^14^N), and sulfur (^34^S/^32^S) were measured by isotope ratio mass spectrometry at Isolab GmbH, Schweitenkirchen, Germany. The average of three measurements per sample was provided and was used for subsequent analyses. Results are reported in δ‐notation in units of per mill (‰) according to the international standards of Vienna Pee Dee Belemnite (V‐PDB) for carbon, Ambient Inhalable Reservoir (AIR) for nitrogen, and Vienna Canyon Diablo Troilite (V‐CDT) for Sulfur. In addition, the laboratory internal standards STD R (collagen from cowhide from the EU project TRACE) and for most samples also STD BRA (collagen from Brasilian cowhide) were reported. Internal analytical errors were recorded as ±0.1‰ for δ^13^C, ±0.2‰ for δ^15^N, and ±0.3‰ for δ^34^S (standard error of the means calculated from 3 or 4 measurements).

We selected samples with a value of >1% collagen portion of dry bone (wt% = amount of extracted collagen/amount of bone powder used for extraction × 100). The molar C:N ratio ([%C/%N] × [14.007/12.011]) in the range of 2.9–3.6 was considered as good quality (Deniro, 1985). As good quality was considered as well, when %C was in the range of 30%–47% and %N in the range of 11%–17.3% (Ambrose, 1990; Van Klinken, 1999). When at least one of the quality criteria was not within the stated range, we excluded the sample from further evaluation. We considered sulfur values when the C/N quality criteria were accepted, and in addition, %S was within the range of 0.15%–0.35%, the C:S ratio between 300 and 900, and the N:S ratio between 100 and 300 (Nehlich & Richards, 2009).

### 2.3 Coding of archeological and paleopathological variables

All individuals were classified according to seven binary variables expressing the presence or absence of specific grave good items. These include: cauldrons, knives, arrowheads (as grave offering), bow implements, and gold spirals. These elements were chosen after a preliminary screening of burial item variability at Tunnug1, and coded as simple absence‐presence in order to maximize sample size. It is important to stress that, for some burials, post‐depositional natural disturbance hampered a safe association between items and individuals. All these cases were not considered in the following analysis.

As mentioned earlier, the presence of perimortem traumas and traces of decapitations in a large number of individuals opens the question about their social status and geographic origin (Milella et al., 2021). We therefore decided to create two additional variables describing the presence‐absence of these features. These two variables overlap in several cases (all decapitated individuals are classified as presenting perimortem trauma, but not all individuals with perimortem lesions are also decapitated) and were analyzed separately.

### 2.4 Statistical protocol

Our analyses include the following steps:

1. We first explored the isotopic variability in the animal and human sample. In order to put Tunnug1 in a broader perspective, we considered for comparison a chosen set of published carbon and nitrogen isotopic ranges from other central Asian contexts (Southern Siberia, Baikal and Western Transbaikalia regions, Kazakhstan, Mongolia) dating to the Bronze, Iron, and Middle Ages (Komarova, 2020; Kradin et al., 2021; Lightfoot et al., 2015; Machicek, 2011; Murphy et al., 2013; Ventresca Miller & Makarewicz, 2019; Weber et al., 2011; Wilkin et al., 2020). Naturally, given the large geographic, chronological, and cultural span represented by these data, we will refer to them only as a broad reference for discussing the patterns observed at Tunnug1.
  We then estimated the dietary contribution of herbivores, freshwater fish, C_4_ and C_3_ plants by means of Bayesian modeling with the software FRUITS (Fernandes et al., 2014). Bayesian modeling was performed using δ^13^C and δ^15^N from all individuals and then excluding individuals younger than 3 years (in order to minimize the trophic effect of breastfeeding on isotopic ratios). Separate models were calculated first without priors, and then assuming as prior a higher dietary contribution of herbivores and C_4_ plants compared with respectively fish and C_3_ plants. The FRUITS models include our isotopic data for humans and herbivores, and published values for freshwater fish, C_3_ and C_4_ plants (Fernandes et al., 2015; Murphy et al., 2013; Svyatko et al., 2007; Varalli et al., 2021). We realize that published data cannot substitute a full isotopic set from the context under study. On the other hand, we think that the obtained results should at least provide some useful information on the dietary contribution of these food resources.
  We then compared the isotope ratios between sexes by means of Mann–Whitney tests, and their variance with a Fligner–Killeen test. We used a Kruskal–Wallis test followed by pairwise Dunn tests to explore differences in isotopic ratios between age classes.
2. We checked for the presence of nonlocal individuals screening for δ^34^S outliers. To this aim, we used the median of sulfur isotopic ratios ± triple the median absolute deviation (3MAD) of herbivores as proxy of local isotopic baseline. Studies on mobility are increasingly using this approach when trying to detect isotopic outliers (Lightfoot & O'Connell, 2016; Milella et al., 2019; Ventresca Miller et al., 2021). The advantage of 3MAD over alternative criteria (e.g., two standard deviations from the mean) is that it offers a more rigorous threshold for the detection of outliers (Leys et al., 2013).
  Due to the possible influence of seasonal herding practices (Vajnštejn, 1980) on herbivores isotope ratios, we also considered an alternative range based on sulfur isotope ratios of humans younger than ca. 3 years (12 individuals, mostly aged below 2 years). We chose neonates and infants since the short lifespan expressed by their collagen isotope ratios should minimize the long‐term effect of seasonal mobility (as expected for a pastoral nomadic population). For simplicity, the ranges estimated from human subadults and herbivores will be henceforth referred to as “Range_subadults” and “Range_herbivores,” respectively.
3. We compared isotope ratios between individuals with and without perimortem traumas by means of Mann–Whitney tests. To this aim, we first considered the presence of perimortem lesions, and then those with clear traces of decapitation (for details see Milella et al., 2021). We then checked for the possible association between funerary features and isotopic ratios while controlling for age and sex using multiple linear regressions.

We performed all analyses using JMP 15.2.0 (SAS Institute 2019) using alpha =0.05.

## 3 RESULTS

### 3.1 Diet: Animals

**FIGURE 2:**
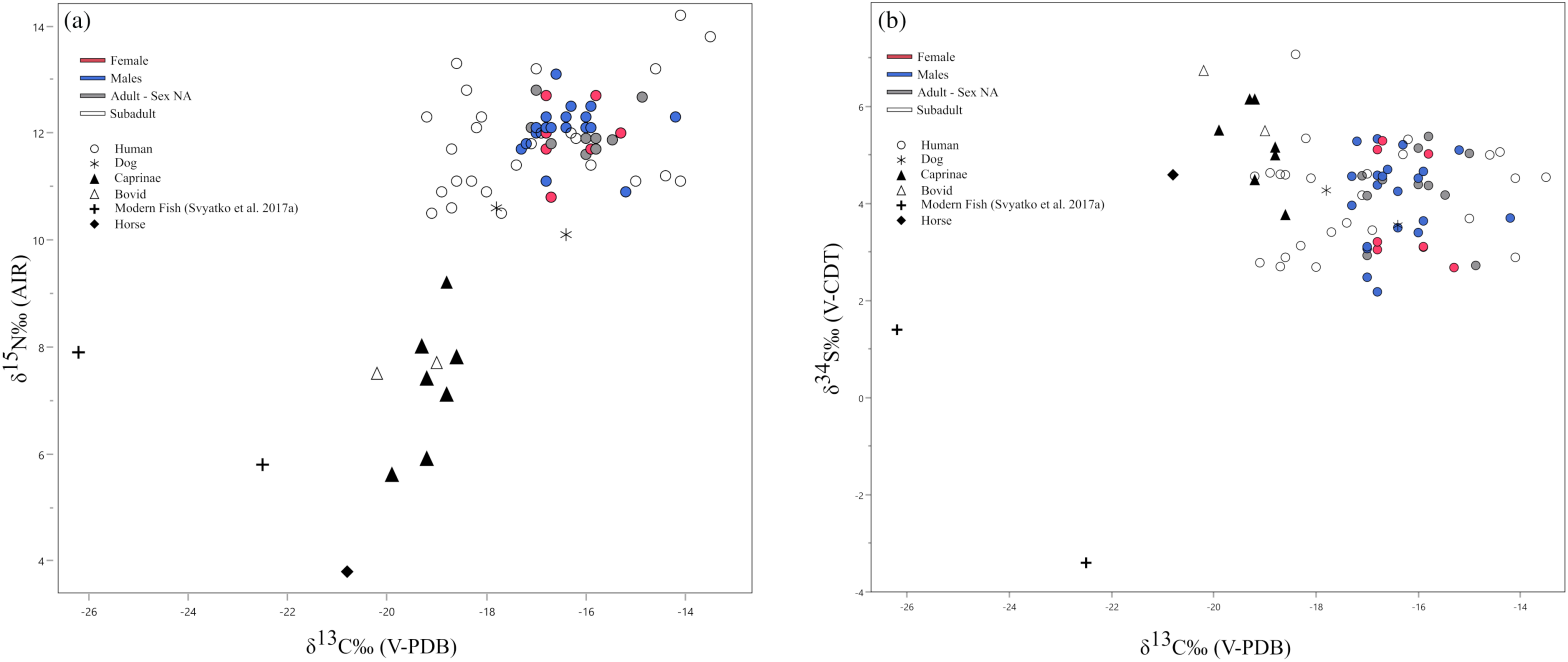
Plots of carbon versus nitrogen (a) and carbon versus sulfur, (b) stable isotope ratios

Table S1 and Figure 2 show the stable isotope ratios of humans and animals from Tunnug1. Figure S1 compares carbon and nitrogen isotope ratios of the individuals fromTunnug1 with the comparative ranges from Bronze Age, Iron Age and Medieval contexts from Central Asia.

With the exception of one sample (Object 44, a post‐Kokel cremation) the collagen in samples fulfilled the quality criteria for biogenic stable carbon, nitrogen and sulfur isotope ratios.

The nine herbivores (seven caprinae and two bovids) show δ^13^C ranging from −20.2‰ to −18.6‰ ($\bar{x}=-19.2\pm 0.5‰)$, and a δ^15^N range between 5.6‰ and 9.2‰ ($\bar{x}=7.4\pm 1.1‰)$. δ^34^S in herbivores range from 3.8‰ to 6.7‰ ($\bar{x}=5.3\pm 0.9)$.

δ^13^C and δ^15^N of the horse (−20.8‰ and 3.8‰, respectively) are depleted compared with those of herbivores, while δ^34^S fall in the range of bovids and caprinae (4.6‰).

The two dogs show carbon isotope ratios of −17.8‰ and − 16.4‰ ($\bar{x}=-17.1\pm 0.99‰)$, and δ^15^N of 10.1‰ and 10.6‰ ($\bar{x}=10.4\pm 0.35‰)$. Dog δ^34^S values are 3.6‰ and 4.3‰ ($\bar{x}=3.9\pm 0.5)$.

Compared with our faunal data, the fish samples published by Svyatko, Polyakov, et al. (2017) presents depleted carbon and sulfur isotope ratios (averages: −24.4‰ and −1.0‰, respectively), and δ^15^N values falling in the range of herbivores (6.9‰).

### 3.2 Diet: Humans

Stable carbon isotope ratios in humans range from −19.2‰ to −13.5‰ ($\bar{x}=-16.6\pm 1.3‰)$, while δ^15^N range from 10.5‰ to 14.2‰ ($\bar{x}=12.0\pm 0.8‰)$. Excluding individuals younger than 3 years of age (due to the trophic level effect associated with breastfeeding –Fuller et al., 2006; Katzenberg et al., 1996), the difference between humans and herbivores in δ^13^C and δ^15^N is 2.8‰ and 4.6‰, respectively. Sulfur isotope ratios range in humans from 2.2‰ to 7.1‰ ($\bar{x}=4.1\pm 0.99‰)$, with a 1.2‰ offset between humans and herbivores.

Carbon stable isotope values of Tunnug1 fall in the range of the comparative Xiongnu and Medieval samples, whereas δ^15^N values fall in the lower range of the other contexts. Considering both isotopes, Tunnug1 plots close to the Mongolian sites of Egiin Gol (Iron Age‐Xiongnu and Ulaanzuukh (Bronze Age) (Figure S1).

We did not find any statistically significant difference in isotope ratios between males and females (Table 2). Average δ^13^C values in males and females are −16.5 ± 0.7‰ and −16.3 ± 0.6‰, respectively, and for δ^15^N of 12.1 ± 0.5‰ and 11.9 ± 0.7‰. Mean δ^34^S values are 3.9 ± 1.2‰ in males and 4.1 ± 0.9‰ in females. In terms of intra‐sex isotopic variance, the latter is higher in females for δ^15^N and δ^34^S, and in males for δ^13^C. These differences are, however, not statistically significant when analyzed with a Fligner–Killeen test.

**TABLE 2:** Results of Mann–Whitney *U* tests and Fligner–Killeen tests between males and females

	Females					Males				Var	*p* (Mann–Whitney *U* test)	*p* (Fligner–Killeen test)
	*n*	Mean	Median	SD		*n*	Mean	Median	SD			
δ^13^C (‰) V‐PDB	7	−16.3	−16.7	0.6	0.4	21	−16.5	−16.7	0.7	0.6	0.3626	0.9563
δ^15^N (‰) AIR	7	11.9	12	0.7	0.4	21	12.1	12.1	0.5	0.2	0.4052	0.3006
δ^34^S (‰) V‐CDT	7	3.9	3.21	1.2	1.3	21	4.1	4.38	0.9	0.8	0.6907	0.7363

Abbreviations: AIR, Ambient Inhalable Reservoir for nitrogen; *N*, number of individuals; SD, standard deviation; Var: variance; V‐CDT, Vienna Canyon Diablo Troilite; V‐PDB, Vienna Pee Dee Belemnite.

**FIGURE 3:**
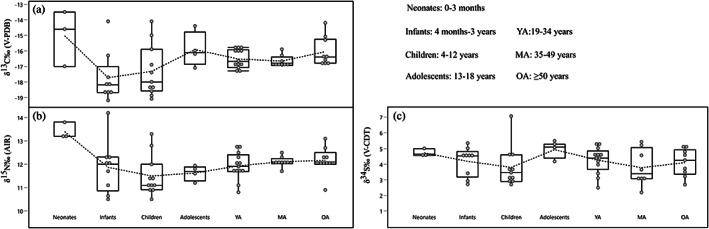
Carbon (a), nitrogen (b), and sulfur (c) stable isotope ratios by age class

Plotting δ^13^C and δ^15^N by age class shows a decrease of both isotope ratios from neonates to infants. In the following age groups δ^13^C is then progressively more enriched. δ^15^N is further depleted in children, showing higher values in adolescents and adults (Figure 3). The effect of age on isotopic patterns is statistically significant for δ^13^C and δ^15^N, but not δ^34^S (Tables 3 and S2). The only statistically significant result from the Dunn tests is the comparison between neonates and children for δ^15^N.

**TABLE 3:** Isotopic values versus age class: results of Kruskal–Wallis tests

		Neonate	Infant	Child	Adolescent	YA	MA	OA	*p* (Kruskal–Wallis test)
δ^13^C (‰) V‐PDB	*n*	3	9	11	3	13	8	10	**0.0229**
	Mean	−15.0	−17.7	−17.3	−15.9	−16.5	−16.7	−16.1	
	Median	−14.6	−18.2	−18.0	−16.2	−16.7	−16.8	−16.4	
	SD	1.8	1.6	1.7	1.4	0.6	0.4	0.9	
δ^15^N (‰) (AIR)	*n*	3	9	11	3	13	8	10	**0.0288** *
	Mean	13.4	11.9	11.5	11.6	11.9	12.1	12.1	
	Median	13.2	12.0	11.1	11.8	11.9	12.1	12.1	
	SD	0.3	1.1	0.9	0.4	0.6	0.2	0.6	
δ^34^S (‰) (V‐CDT)	*n*	3	9	11	3	13	8	10	0.4203
	Mean	4.7	4.2	3.8	4.9	4.2	3.8	4.2	
	Median	4.6	4.5	3.5	5.1	4.4	3.4	4.4	
	SD	0.2	0.9	1.3	0.6	0.8	1.1	0.9	

Abbreviations: AIR, Ambient Inhalable Reservoir for nitrogen; *N*, number of individuals; SD, standard deviation; V‐CDT, Vienna Canyon Diablo Troilite; V‐PDB, Vienna Pee Dee Belemnite.

* Significant post‐hoc Dunn test: Neonates versus children (*p*: 0.0218).

Estimates of dietary contribution provided by FRUITS (Figure S2 and Table S3) confirm a large contribution of C_4_ plant products to human diet (mean estimate: 49.6%–53%), followed by herbivores‐derived foodstuffs (mean estimate: 21.7%–30.2%) and to a minor extent C_3_ plants and fish.

### 3.3 Regional mobility

**FIGURE 4:**
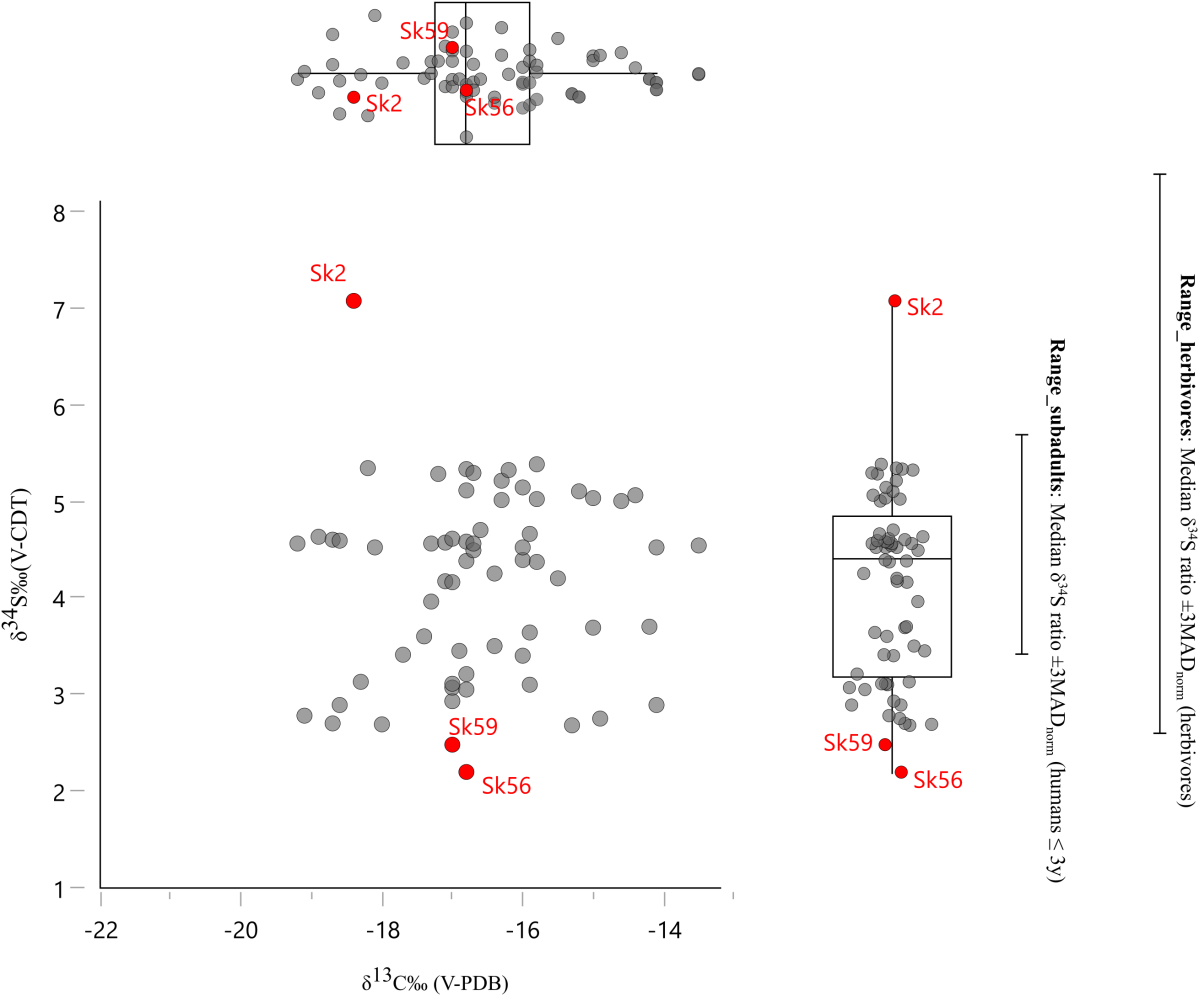
Plots of carbon versus sulfur stable isotope ratios with highlighted the sulfur Range_subadults and Range_herbivores and, in red, the isotopic outliers

In Figure 4, we plot the δ^34^S and δ^13^C of humans, their spread, as well as Range_subadults and Range_herbivores. These two ranges substantially differ, with obvious influences on the resulting number of potential outliers. Several individuals fall outside the lower limit of Range_subadults, and one individual (skeleton 2, a child of 6–8 years) is outside its upper limit. Conversely, only two individuals fall outside the lower limit of Range_herbivores: skeleton 56 (middle adult male) and 59 (young adult male).

### 3.4 Isotopic distribution versus trauma and funerary treatment

**FIGURE 5:**
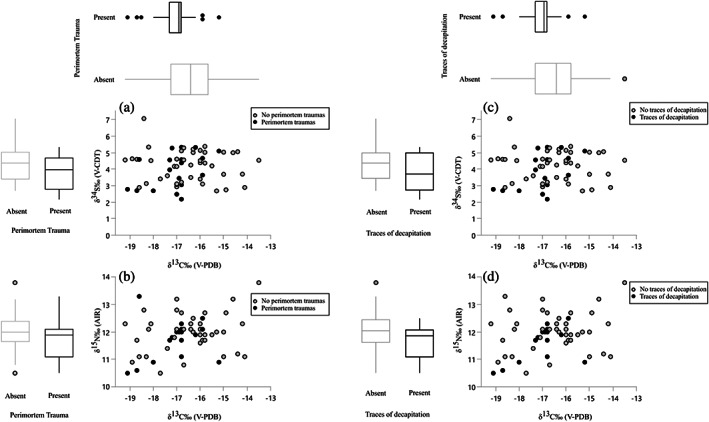
Plots of carbon versus nitrogen and carbon versus sulfur stable isotope ratios with highlighted in black individuals presenting perimortem traumas (a, b) and traces of decapitation (c, d)

Statistically significant differences between individuals with and without perimortem traumas are found in adults for δ^13^C. Individuals with perimortem lesions and traces of decapitation show in average more depleted carbon isotope ratios (Table 4 and Figure 5). Marginally non‐significant is also the difference in δ^15^N between adults with and without perimortem lesions. Also, in this case individuals with trauma show more depleted δ^15^N. No statistically significant association was found between presence of skeletal lesions/decapitation and δ^34^S. Interestingly, the two individuals with the most depleted δ^34^S value (adult males skeletons 56 and 59) show also traces of decapitation.

**TABLE 4:** Isotopic values versus presence of perimortem traumas and traces of decapitation: results of Mann–Whitney *U* tests

(a)	Perimortem trauma								
	No				Yes				*p* (Mann–Whitney *U* test)
	*n*	Mean	Median	SD	*n*	Mean	Median	SD	
All individuals									
δ^13^C (‰) V‐PDB	41	−16.5	−16.4	1.4	19	−17.1	−16.9	1.0	0.07
δ^15^N (‰) AIR	41	12.0	12.0	0.7	19	11.7	11.9	0.7	0.17
δ^34^S (‰) V‐CDT	41	4.2	4.4	0.9	19	3.9	4.0	1.1	0.31
Adults only									
δ^13^C (‰) V‐PDB	23	−16.1	−16.0	0.7	13	−16.7	−16.8	0.6	0.01
δ^15^N (‰) AIR	23	12.1	12.1	0.5	13	11.8	12.0	0.4	0.11
δ^34^S (‰) V‐CDT	23	4.1	4.3	0.9	13	4.0	4.4	1.1	0.87

(b)	Traces of decapitation								
	No				Yes				*p* (Mann–Whitney *U* test)
	*n*	Mean	Median	SD	*n*	Mean	Median	SD	
All individuals									
δ^13^C (‰) V‐PDB	44	−16.5	−16.4	1.4	16	−17.1	−17.0	1.0	0.09
δ^15^N (‰) AIR	44	12.0	12.1	0.7	16	11.7	11.9	0.6	0.08
δ^34^S (‰) V‐CDT	44	4.2	4.4	0.9	16	3.8	3.7	1.1	0.28
Adults only									
δ^13^C (‰) V‐PDB	25	−16.1	−16.0	0.7	11	−16.7	−16.8	0.6	0.01
δ^15^N (‰) AIR	25	12.1	12.1	0.5	11	11.9	12.0	0.4	0.21
δ^34^S (‰) V‐CDT	25	4.1	4.3	0.9	11	4.0	4.6	1.1	0.97

Abbreviations: AIR, Ambient Inhalable Reservoir for nitrogen; *N*, number of individuals; SD, standard deviation; V‐CDT, Vienna Canyon Diablo Troilite; V‐PDB, Vienna Pee Dee Belemnite.

We did not find any statistically significant association between funerary variables and isotopic values (Table S4).

## 4 DISCUSSION

Our study aimed to test three main hypotheses about diet, mobility, and their link to social differentiation (or lack thereof) among the individuals buried at Tunnug1. In the following sections, we consider each hypothesis separately, and discuss their compatibility with the observed data as well as the limitations of our analyses.

### 4.1 Diet

Our first hypothesis postulated a mixed subsistence economy for the community of Tunnug1, and a diet incorporating both animal proteins and other resources. Results largely confirm these prediction.

The δ^13^C range of herbivores is consistent with a diet dominated by C_3_ plants, with a minor inclusion of C_4_ resources (e.g., wild C_4_ plants and/or inclusion in the fodder of straw from millet) or access to pastures with variable aridity (Flohr et al., 2011). The latter scenario, in conjunction with the possible presence of suckled individuals, would explain the wide range of δ^15^N of herbivores. Compared with caprinae and bovids, the horse shows depleted carbon and nitrogen isotope ratios, as expected due to their different digestive systems (see above). δ^13^C and δ^15^N in the two dogs suggest a diet featuring a relatively large proportion of plant foods, possibly from human food waste (cf. Albizuri et al., 2021; Laffranchi et al., 2016; Meadows et al., 2020).

Human δ^13^C values are consistent with a mixed diet including C_3_ and C_4_ resources (Schoeninger & Moore, 1992). The offset in δ^15^N between herbivores and humans (4.6‰) points to a substantial consumption of terrestrial animal proteins (via meat and/or dairy products). δ^13^C values in collagen tend to be enriched of ca. 0.8‰–1‰ by trophic level (Lee‐Thorp et al., 1989; Styring et al., 2017). This, in conjunction with the C_3_‐based diet of herbivores suggests that the enriched δ^13^C in humans derive from the direct consumption of C_4_ foodstuffs (most likely millet), rather than via animal products.

Various studies indicate that Eurasian steppe populations frequently included freshwater fish in their diet (Murphy et al., 2013; Privat et al., 2007; Shishlina et al., 2007; Zhang et al., 2015). δ^15^N values in modern fish fall in the range of the herbivores from Tunnug1. This makes it impossible to evaluate a contribution of freshwater resources to human diet based on nitrogen isotope ratios. However, the strongly depleted carbon and especially sulfur isotope ratios of modern fish compared with those of the “archeological” herbivores would indicate that freshwater resources did not substantially contribute to the human diet. In the opposite case, considering an isotopic enrichment for trophic level of ca. 0.8%–1‰ for carbon (Styring et al., 2017) and ≤1 ‰ for sulfur (Nehlich, 2015), we would expect δ^13^C and δ^34^S in humans to be much more depleted than observed here. Nonetheless, a partial consumption of freshwater fish can be (cautiously) proposed for at least some of the individuals showing particularly depleted carbon and sulfur isotope ratios. It is important to point out that δ^13^C, δ^15^N, and δ^34^S values in freshwater environments are quite variable (Katzenberg & Weber, 1999; Nehlich, 2015; Privat et al., 2007; Svyatko, 2016; Svyatko, Reimer, & Schulting, 2017). It is therefore unclear to which extent the data from Svyatko, Polyakov, et al. (2017) (which represent contexts chronologically and geographically different from ours) can be used as a reference for Tunnug1. Missing archaeozoological remains of fish from Tunnug1, a planned sampling of freshwater fish from the region may help clarifying this issue.

FRUITS estimates for the contribution of C_4_ resources to human diet are quite high. A previous estimate of dietary contribution of millet at Iron Age Aymyrlyg (5th–2nd c. BCE) and Ai‐Dai (8th–3rd c. BCE) is 35 ± 10% (Murphy et al., 2013). However, as already suggested by these authors, this estimate which is based on a simple linear mixing model is probably conservative. This is also suggested by the results of FRUITS for Tunnug1, a context showing δ^13^C values depleted compared with Aymyrlyg and Ai‐Dai. The identification of the C_4_ resource with millet (*Panicum* sp.) at Tunnug1 is supported by the use of this crop in Southern Siberia as early as 2000 BCE (Ventresca Miller & Makarewicz, 2019) and by environmental data and economic reasons. First, wild C_4_ grasses (e.g., *Chenopodium* spp.) are a minor component of steppe biome when compared with C_3_ ones (Liu et al., 2004; Pyankov et al., 2000; Wang, 2003; Wilkin et al., 2020). The exploitation of millet in Tuva has been also documented for the site of Kokel (Kenk, 1984; Vajnštejn & Dyakonova, 1966: 259, 263, 265), and ethnologically (Vajnštejn, 1980: 145–165). Second, millet is a crop well‐suited for cultivation in the steppe, due to its drought tolerance, short growing season and high yield per plant (Miller et al., 2016; Wang et al., 2016). Millet may also have arrived in the region around Tunnug1 via trade, as a complementary source to local farming. This hypothesis, however, would badly fit with our estimates of a diet largely based on this food resource. Rather, local production through farming seems to be a better explanation for our data.

The lack of differences in isotopic values between males and females indicate their access to the same or largely similar dietary resources. This would agree with the results of previous studies on populations geographically close by (Murphy et al., 2013; Svyatko et al., 2013). We cannot exclude the presence of sex‐specific dietary customs, which would be isotopically undetectable (e.g., different cuts of meat). Subtle dietary differences between sexes may moreover underlie the larger variance of δ^15^N in females. However, this variance does not significantly differ from that of males, and physiological stress can influence δ^15^N values (D'Ortenzio et al., 2015). A larger variance in δ^15^N may therefore be the result of the presence of individuals with variable health conditions combined with the small size of the female sample.

The variation of δ^15^N values throughout age classes (Figure 2b) is consistent with a trophic effect of breastfeeding in neonates and infants possibly extended until 2–3 years of age, followed by the gradual process of weaning (Laffranchi et al., 2018). The differences between neonates and infants' carbon isotope ratios (Figure 3a) may be explained by a combination of dietary influences. Enriched δ^13^C values could be related to breastfeeding (Siebke et al., 2019). Considering the relatively small trophic effect of δ^13^C (circa 1‰ – Fuller et al., 2006), if mothers had a high proportion of C_4_ resources in their diet, their enriched isotope ratios will then be passed on to the breastfed offspring. The gradual incorporation of other food resources in the diet of older infants (e.g., freshwater foodstuffs, C_3_ plants and/or dairy or meat products from mammals feeding on C_3_ plants) could explain the depleted stable carbon and sulfur isotope ratios observed in this and the children age classes when compared with neonates (Figure 3c).

### 4.2 Mobility

Our second hypothesis postulated that mobility at Tunnug1 was relatively low and represented by few nonlocal individuals. Data on sulfur agrees with the first of these expectations.

It needs to be mentioned, however, that our discussion of mobility at Tunnug1 is complicated by three main issues:

1. The lack of sulfur isoscapes for the region of Tuva and surrounding areas. This means that isotopic convergence of different regions would mask the presence of nonlocals in our sample. Moreover, the lack of local isoscapes makes it difficult to evaluate the degree (short vs. long distance) of mobility of potential nonlocals.
2. The mixed subsistence economy of this population, which likely included seasonal movements of people and herds (Khazanov, 1978; Vajnštejn, 1980). As a result, the presence of nonlocals may be hidden by the “mobile” isotopic signature of humans and animals. This consideration is however counterbalanced by the sulfur values of animals, which tend to cluster together. This in turn suggests that even if such seasonal movements took place, they were in any case limited inside an isotopically homogenous area.
3. The contribution of freshwater resources to human diet, which, even if minor, would have affected human δ^34^S variability. This is especially important when considering the depleted isotope ratios characterizing several individuals at Tunnug1.

Although various individuals fall outside Range_subadults, only two are found outside the larger Range_herbivores: Skeletons 56 and 59. For some of the analyzed individuals depleted δ^34^S values may result from the consumption of freshwater fish. In the case of Skeletons 56 and 59, however, this explanation seems difficult to accept based on their carbon isotopic signature. With a substantial consumption of freshwater fish, we would indeed expect more depleted δ^13^C values for these individuals if we consider the fish data from Svyatko, Polyakov, et al. (2017).

Another potential nonlocal is Skeleton 2, which falls in the upper sulfur range of herbivores, but is sharply different from the other humans for its enriched isotopic signature (Figure 4).

In sum, a parsimonious approach, which also takes into account the possible effect of freshwater fish consumption on sulfur isotope ratios, would identify the three individuals 2, 56, and 59 as possible nonlocals.

A number of publications have focused on prehistoric mobility in the Eurasian steppe, including populations from Kazakhstan (Bernbeck et al., 2011; Ventresca Miller et al., 2017), Baikal region (Haverkort et al., 2008; Weber & Goriunova, 2013), Mongolia (Machicek et al., 2019), the Carpathian Basin (Gerling et al., 2012), and the Pontic region (Ventresca Miller et al., 2019, 2021). Overall, these studies suggest that, with few exceptions, mobility among Eurasian pastoralists was mostly small‐scale and within limited ranges (Makarewicz, 2018). The small number of isotopic outliers at Tunnug1 suggests a limited mobility in this population, a result that adds new data on mobility among Eurasian populations. Most of the above studies are focused on Bronze and Iron Age populations, with only few data for more recent contexts (Machicek et al., 2019). The isotopic patterns highlighted at Tunnug1 would suggest the maintenance of similar subsistence strategies through time. However, one needs to consider the partial nature of our data, which allow only broad‐brush reconstruction, and the local economic and cultural variability which is probably hidden underneath the observed similarities.

The lack of isoscapes for Tuva and surrounding regions, and the variability of δ^34^S in rocks (Nehlich, 2015), do not allow specific hypotheses about the geographic origin of the nonlocal individuals, and about their long versus short distance mobility. The geology of the areas surrounding Tunnug1 raises the possibility of isotopic convergence (similar geologic settings in different areas).

### 4.3 Social correlates of diet

Our third hypothesis postulated a lack of substantial differences in diet between sexes, and a lack of correlation between diet and social differentiation. Our results only partially confirm these expectations: whereas isotopic data do not differ between contrasting funerary treatments, their association with perimortem trauma raises intriguing questions about the diet and, possibly, geographic origin of decapitated individuals.

As mentioned, funerary variability at Tunnug1 is rather homogenous, including few luxury goods and no burials suggesting an exceptionally high social status of the deceased (Milella et al., 2021; Sadykov et al., 2021). The comparison of funerary and isotopic data (Table S4) further suggests the absence of sharp social differences at the site possibly responsible of different dietary habits. It is important to stress that subtle dietary differences may be left undetected isotopically (see above), and that our funerary classification, being rather rough, is likely to miss nuanced social differences (cf. Laffranchi et al., 2019; Milella et al., 2019).

Moreover, it remains questionable to which extent the individuals from Tunnug1 are representative of their original population. The proximity of the Kokel cemetery to a royal Iron Age kurgan raises the possibility that this location was charged with a specific symbolic meaning. Cultural (possibly status‐related) factors may have led the choice to bury some individuals here. If that was the case, we may well be dealing with just a section of the original population, and this may explain the homogenous funerary treatment and dietary behavior.

About the relationship between trauma lesions and isotopic values, the depleted carbon and (to a lesser extent) nitrogen isotope ratios of the individuals with trauma deserve some attention (Table 4 and Figure 5), since it may hint at a specific diet of these individuals, and, in particular, at a lower consumption of millet and animal proteins. If this was related to their specific (potentially lower) social standing is however difficult to postulate. Depleted δ^13^C values may also signal the access by these individuals to a different biome, and, indirectly, their nonlocal origin (Eerkens et al., 2014; Hakenbeck et al., 2010). A possible association between trauma and mobility may also be cautiously proposed based on sulfur isotope ratios. Although no statistical difference separates individuals with and without trauma regarding δ^34^S values, it is interesting to note that two of the possible nonlocal individuals (Sk 56 and 59) present traces of decapitation. As already mentioned, the lack of more substantial patterns could in this case be explained by the geological variability of Southern Siberia, and isotopic convergence between different areas.

Even assuming a partial association between trauma and individual nonlocal origin, the cultural significance of this link remains puzzling. The type, anatomical distribution, and demographic patterns of perimortem lesions at Tunnug1 suggest their link to combats and/or executions (Milella et al., 2021), but open questions relate to the dynamics (raids against or from other groups) and actors involved in these violent events, and the relationships between the individuals buried at Tunnug1 (same vs. different groups, familiar groups, etc.). Further analyses (e.g., paleogenetic investigations of kinship) may provide additional insights on at least some of these points.

## 5 CONCLUSION

Isotopic studies of the Eurasian steppe populations are for the most part focused on the Bronze and Iron Age contexts. This body of research has suggested that the subsistence economies and lifestyles of steppe societies was more variable than once suspected. Our study complements previous research by adding new insights about the exploitation of different dietary resources (millet, herbivores, and to a minor extent freshwater fish) and degrees of regional mobility for a rarely investigated geochronological context (Southern Siberia between the 2nd–4th centuries CE). Millet was an important dietary source for the people buried at Tunnug1. Regional mobility was only limited, although further data on local isoscapes are needed to better contextualize our results. Archeological and isotopic data agree in depicting a community lacking sharp social differences.

Additional sampling of local fauna (especially freshwater fish) and the inclusion of oxygen and strontium isotopic data may help to address a number of research questions left open by this study.

## CONFLICT OF INTEREST

The authors declare that they have no conflict of interest.

## AUTHOR CONTRIBUTIONS

**Marco Milella:** Conceptualization (lead); data curation (equal); formal analysis (lead); investigation (equal); software (lead); visualization (lead); writing – original draft (lead); writing – review and editing (lead). **Gino Caspari:** Conceptualization (equal); funding acquisition (equal); investigation (equal); project administration (lead); resources (lead); writing – original draft (equal); writing – review and editing (equal). **Zita Laffranchi:** Conceptualization (equal); data curation (equal); formal analysis (equal); investigation (equal); methodology (equal); writing – original draft (equal); writing – review and editing (equal). **Gabriele Arenz:** Data curation (equal); investigation (equal); methodology (equal); writing – original draft (equal); writing – review and editing (equal). **Timur Sadykov:** Conceptualization (equal); funding acquisition (equal); investigation (equal); project administration (lead); resources (lead); writing – original draft (equal); writing – review and editing (equal). **Jegor Blochin:** Conceptualization (equal); funding acquisition (equal); investigation (equal); project administration (lead); resources (lead); writing – original draft (equal); writing – review and editing (equal). **Marcel Keller:** Writing – original draft (equal); writing – review and editing (equal). **Yulija Kapinus:** Writing – review and editing (equal). **Sandra Lösch:** Conceptualization (equal); data curation (equal); funding acquisition (lead); investigation (equal); methodology (equal); resources (lead); writing – original draft (equal); writing – review and editing (equal).

## Supporting information


**Figure S1** Plot of carbon and nitrogen stable isotope ratios showing the mean and standard deviation of Tunnug1 (gray triangle) compared with published values for other Central Asian contexts dating from the Bronze Age to Medieval times.

**Figure S2** FRUITS estimates of dietary contribution for C_3_ and C_4_ plants, herbivores, and freshwater fish. Upper row: estimates calculated without priors for all individuals (a) and only individuals older than 3 years (b). Lower row: estimates obtained assuming a higher contribution of C_4_ compared with C_3_ plants and of herbivores compared with freshwater fish for all individuals (c) and individuals older than 3 years (d).

**Table S1** Anthropological, isotopic, and archeological datasets.

**Table S2** Post‐hoc Dunn tests for isotopic differences between age classes (see also Table 3).

**Table S3** Bayesian modeling of dietary compositions using FRUITS. Data entry and results.

**Table S4** Results of multiple linear regression used to test for associations between funerary and isotopic data. df, degrees of freedom.

## Data Availability

The data that supports the findings of this study are available in the supplementary material of this article.
