# Supplementary material for: Dining in Tuva: Social correlates of diet and mobility in Southern Siberia during the 2nd–4th centuries CE
Source: Am J Biol Anthropol. 2022 Mar 7;178(1):124–39. doi: 10.1002/ajpa.24506 (PMC9314596; doi:10.1002/ajpa.24506)
Supplement: Supplementary file 1 — Figure S1 Plot of carbon and nitrogen stable isotope ratios showing the mean and standard deviation of Tunnug1 (gray triangle) compared with published values for other Central Asian contexts dating from the Bronze Age to Medieval times. [file AJPA-178-124-s003.pdf]

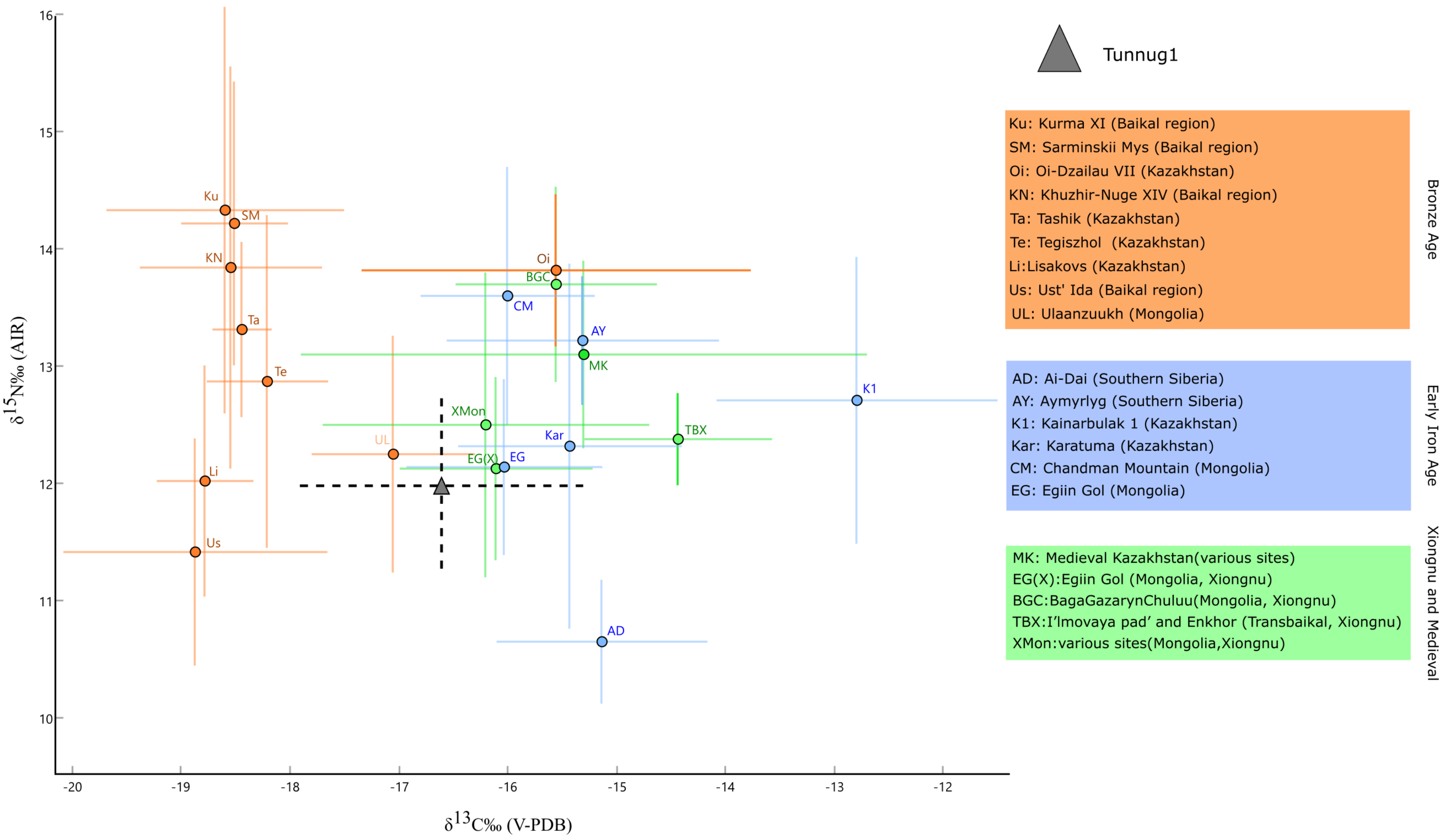

Tunnug1
Ku: Kurma XI (Baikal region)
SM: Sarminskii Mys (Baikal region)
Oi: Oi-Dzailau VII (Kazakhstan)
KN: Khuzhir-Nuge XIV (Baikal region)
Ta: Tashik (Kazakhstan)
Te: Tegiszhol (Kazakhstan)
Li:Lisakovs (Kazakhstan)
Us: Ust' Ida (Baikal region)
UL: Ulaanzuukh (Mongolia)
Bronze Age
AD: Ai-Dai (Southern Siberia)
AY: Aymyrlyg (Southern Siberia)
K1: Kainarbulak 1 (Kazakhstan)
Kar: Karatuma (Kazakhstan)
CM: Chandman Mountain (Mongolia)
EG: Egiin Gol (Mongolia)
Early Iron Age
MK: Medieval Kazakhstan(various sites)
EG(X):Egiin Gol (Mongolia, Xiongnu)
BGC:BagaGazarynChuluu(Mongolia, Xiongnu)
TBX:I'lmovaya pad' and Enkhor (Transbaikal, Xiongnu)
XMon:various sites(Mongolia,Xiongnu)
Xiongnu and Medieval
Ku
SM
KN
Ta
Te
Li
Us
UL
Oi
BGC
CM
AY
MK
XMon
EG(X)
EG
Kar
TBX
K1
AD
δ15N‰ (AIR)
δ13C‰ (V-PDB)
16
15
14
13
12
11
10
-20
-19
-18
-17
-16
-15
-14
-13
-12
