# Supplementary figures and images for: Dining in Tuva: Social correlates of diet and mobility in Southern Siberia during the 2nd–4th centuries CE
Source: Am J Biol Anthropol. 2022 Mar 7;178(1):124–39. doi: 10.1002/ajpa.24506 (PMC9314596; doi:10.1002/ajpa.24506)

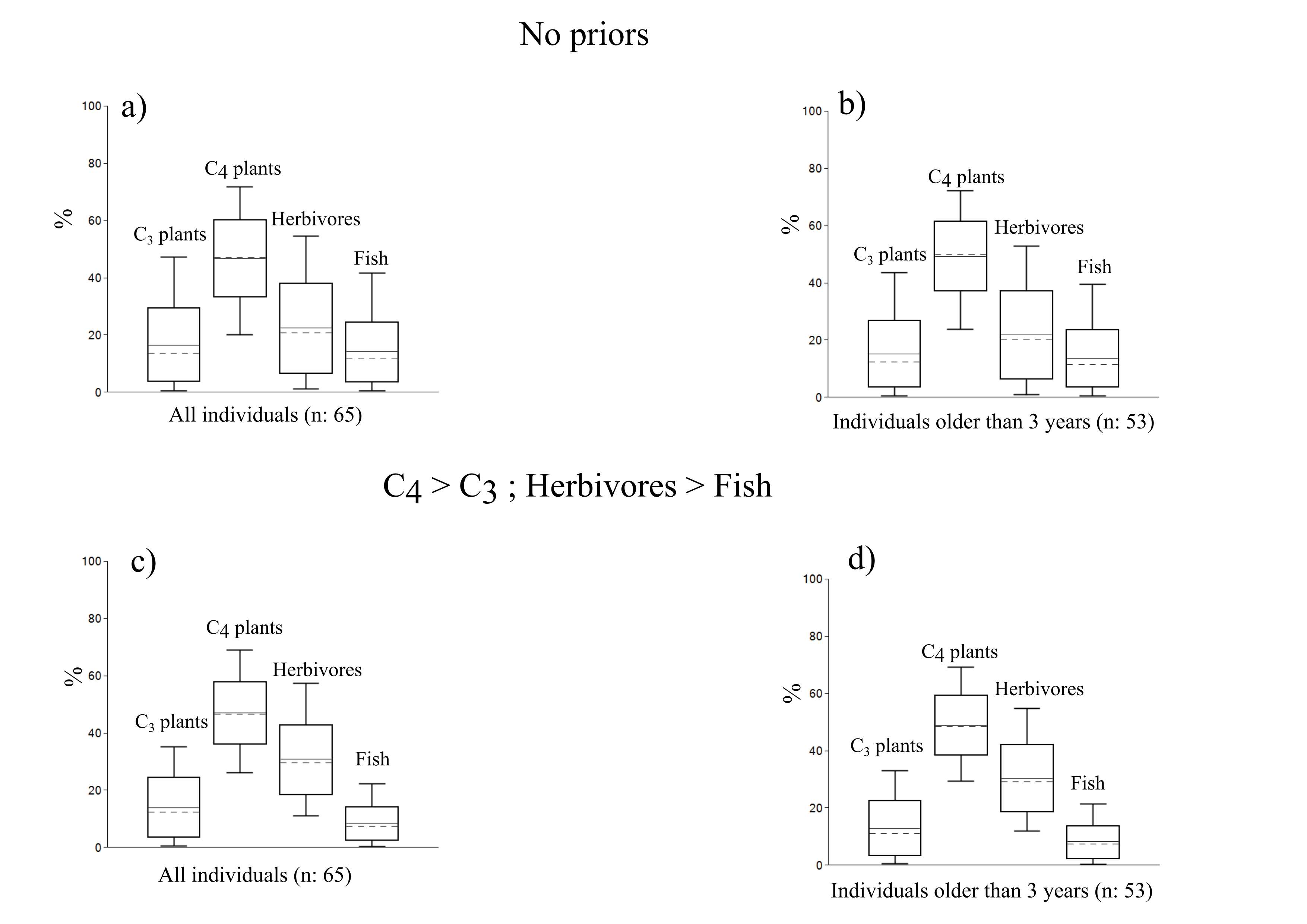

Supplement: Supplementary file 2 — Figure S2 FRUITS estimates of dietary contribution for C3 and C4 plants, herbivores, and freshwater fish. Upper row: estimates calculated without priors for all individuals (a) and only individuals older than 3 years (b). Lower row: estimates obtained assuming a higher contribution of C4 compared with C3 plants and of herbivores compared with freshwater fish for all individuals (c) and individuals older than 3 years (d). [file AJPA-178-124-s004.tif]
